# GQDs-MSNs nanocomposite nanoparticles for simultaneous intracellular drug delivery and fluorescent imaging

**DOI:** 10.1007/s11051-018-4416-y

**Published:** 2018-11-17

**Authors:** Dorota Flak, Łucja Przysiecka, Grzegorz Nowaczyk, Błażej Scheibe, Mikołaj Kościński, Teofil Jesionowski, Stefan Jurga

**Affiliations:** 1 NanoBioMedical Centre, Adam Mickiewicz University in Poznań, Umultowska 85, 61-614 Poznań, Poland; 2 Department of Physics and Biophysics, Poznań University of Life Sciences, Wojska Polskiego 38/42, 60-637 Poznań, Poland; 3 Institute of Chemical Technology and Engineering, Faculty of Chemical Technology, Poznan University of Technology, Berdychowo 4, 60-965 Poznań, Poland

**Keywords:** Graphene quantum dots, Mesoporous silica nanoparticles, Nanocarriers, Cellular uptake, Drug delivery, Real-time monitoring of drug release, Bioimaging, Nanomedicine

## Abstract

Although number of stimuli-responsive drug delivery systems based on mesoporous silica nanoparticles (MSNs) have been developed, the simultaneous real-time monitoring of carrier in order to guarantee proper drug targeting still remains as a challenge. GQDs-MSNs nanocomposite nanoparticles composed of graphene quantum dots (GQDs) and MSNs are proposed as efficient doxorubicin delivery and fluorescent imaging agent, allowing to monitor intracellular localization of a carrier and drug diffusion route from the carrier.

Graphene quantum dots (average diameter 3.65 ± 0.81 nm) as a fluorescent agent were chemically immobilized onto mesoporous silica nanoparticles (average diameter 44.08 ± 7.18 nm) and loaded with doxorubicin. The structure, morphology, chemical composition, and optical properties as well as drug release behavior of doxorubicin (DOX)-loaded GQDs-MSNs were investigated. Then, the in vitro cytotoxicity, cellular uptake, and intracellular localization studies were carried out. Prepared GQDs-MSNs form stable suspensions exhibiting excitation-dependent photoluminescence (PL) behavior. These nanocomposite nanoparticles can be easily DOX-loaded and show pH- and temperature-dependent release behavior. Cytotoxicity studies proved that GQDs-MSNs nanocomposite nanoparticles are nontoxic; however, when loaded with drug, they enable the therapeutic activity of DOX via its active delivery and release. GQDs-MSNs owing to their fluorescent properties and efficient in vitro cellular internalization via caveolae/lipid raft-dependent endocytosis show a high potential for the optical imaging, including the simultaneous real-time optical tracking of the loaded drug during its delivery and release.

**Electronic supplementary material:**

The online version of this article (10.1007/s11051-018-4416-y) contains supplementary material, which is available to authorized users.

**Graphical abstract:**
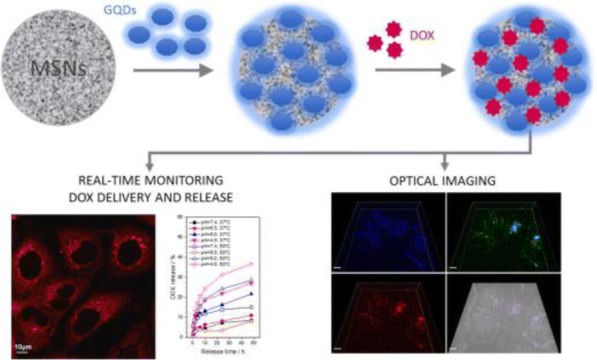
ᅟ

## Introduction

The development of theranostic nanoplatforms for simultaneous diagnosis and therapy, particularly in cancer treatment, is enabled by the combination of nanomaterials with different functionalities. Among a variety of multifunctional nanoplatforms, mesoporous silica-based nanostructures and nanocomposite materials has been recognized and widely studied as promising drug carriers owing to their mesoporous structure, their unique properties, such as large surface area and pore volume, high chemical stability, reactive surface, but also cell membrane-penetration ability and low cytotoxicity (Lee et al. 2011). Mesoporous silica nanoparticles (MSNs) have been employed so far as an efficient carrier of various therapeutics, but also imaging agents, including other functional nanostructured materials. It was proposed that MSNs-based drug delivery systems facilitate controlled delivery and release of anticancer drugs, thus enhancing their therapeutic efficiency along with diminishing their side effects in comparison to standard drug administration (Bharti et al. 2015). Up to date, many MSNs-based theranostic nanoplatforms for bioimaging, drug delivery, and therapy have been developed, taking the advantage of different nanoparticles as capping agents, such as, e.g., Fe_3_O_4_ (Lee et al. 2010), Au (Ma et al. 2012), CdS (Lai et al. 2003), or embedded into MSNs as a core, e.g., Fe_3_O_4_ (Yao et al. 2017a, b), but also paramagnetic ions (Gd^3+^, Mn^2+^) incorporation (Lin et al. 2004) or their chelates (Cao et al. 2015). Another approach also includes the advantage of different release stimuli, such as pH, redox potential, adenosine triphosphate gradient, enzymes, temperature, and multi-stimuli-responsive systems (Moreira et al. 2016).

Number of developed stimuli-responsive drug delivery systems based on MSNs have been developed so far; however, the simultaneous real-time monitoring of the drug carrier in order to guarantee proper drug targeting remains as a challenge. Despite the great potential of MSNs as an efficient drug carrier, these nanoparticles cannot itself emit a fluorescence signal allowing for their detection, however not only when the drug has been released, but also before the release, allowing to monitor intracellular localization of a carrier and diffusion route of the drug from the carrier. This issue has been addressed by the fluorescent labeling of MSNs by capping or encapsulation with fluorescence organic dyes, e.g., fluorescein isothiocyanite (Lu et al. 2009) up-conversion nanoparticles (Niu et al. 2014) and more recently with quantum dots (QDs) (Zhang et al. 2016; Yao et al. 2017a).

QDs are fluorescent nanocrystals, which are considered as an ideal fluorescent agent for bioimaging owing to their many superior properties compared to organic dyes (Resch-Genger et al. 2008). These properties are photostability, broad excitation wavelength, narrow emission, continuous and broad absorption spectra, and finally susceptibility to surface functionalization, including biomolecules. The most commonly used so far QDs contain heavy metals (e.g., CdHgTe, CdTeSe@CdZnS, CdSe@ZnS), which cause undesirable biological and environmental effects, and thus limit their use in biological applications. Graphene quantum dots (GQDs) have emerged as an alternative and a new class of QDs with fluorescent properties (Wen et al. 2015). GQDs are kind of zero-dimensional small graphene sheets fragments, in which the electronic transport is confined in three spatial dimensions. Due to the quantum confinement and edge effects, GQDs possess a non-zero band-gap, and therefore emit luminescence upon the excitation. GQDs are built-up from carbon, which is abundant in the biological systems; therefore, they are considered as a biocompatible nontoxic material. Moreover, GQDs show a molecule-like character and contain a number of carboxylic, epoxy and hydroxyl groups; therefore, they can be easily dissolved in water-based solvents and are easy for further functionalization. Finally, GQDs exhibit stable photoluminescence and superior resistance to photobleaching in comparison to traditional semiconductor QDs and organic dyes. Yao et al. (Yao et al. 2017a, b) reported on the GQDs as caps and local photothermal generators and magnetic mesoporous silica nanoparticles as drug carriers and magnetic thermoseeds, which exhibited strong synergetic effect, resulting in high efficiency to kill cancer cells. Later, Yao et al. (Yao et al. 2017b) reported on GQDs-capped MSNs with a potential for combined chemo- and photothermal cancer therapy, showing pH- and temperature-responsive doxorubicin release, and NIR-induced photothermal cytotoxicity. A similar system has been reported by Huang et al. (Huang et al. 2016), where GQDs-decorated MSNs have been prepared through the electrostatic interaction in contrast to previously mentioned studies, and have been successfully used for aspirin loading and its release. The imaging capacity of GQDs/MSNs system next to the drug delivery has been reported for the first time by Chen et at. (Chen et al. 2014), where GQDs were capped onto MSNs through an acid-cleavable acetal bond, and hence the acidic pH-triggered doxorubicin release from mesopores.

Summarizing, taking the advantage of both components properties, the combination of MSNs and GQDs may provide a new strategy for integrated nanocomposite system for an efficient optical bioimaging and drug delivery system, which is yet to be developed.

In order to address current challenges concerning the theranostic nanoplatform, such as increase of the efficient uptake and accumulation in cancer cells, effective drug delivery, and controlled release with simultaneous imaging and real-time monitoring capability, GQDs-MSNs nanocomposite nanoparticles were prepared by the immobilization of GQDs onto MSNs, which were then loaded with doxorubicin as a model anticancer drug. The release studies were performed in pH- and temperature-dependent manner. So far, most of the GQDs/MSNs-integrated nanoplatforms have been studied mainly for controlled drug delivery, and also for other therapeutic modalities, but their potential as bioimaging agent has been rather underestimated and dominated by therapeutic activities of the system. Therefore, further in this manuscript, the focus is on the detailed investigation on the cellular uptake and intracellular translocation of the GQDs-MSNs, which are the basis for the possibility of the simultaneous imaging and real-time monitoring not only of the drug but also the drug carrier, which has not been well presented until now. The efficiency of prepared GQDs-MSNs nanocomposite nanoparticles for the simultaneous drug delivery and release next to the bioimaging was evaluated using HeLa cancer cells as a model cellular system.

## Experimental

### Materials and methods

Sodium citrate tribasic dehydrate (NaCitr, ≥ 99%), thiourea (TU, ≥ 99%), tetraethyl orthosilicate (TEOS, 98%), hexadecyltrimethylammonium bromide (CTAB, ≥ 99%), N-(3-dimethylaminopropyl)-N′-ethylcarbodiimide hydrochloride (EDC), (3-aminopropyl) triethoxysilane (APTES, ≥ 98%), and phosphate-buffered saline (PBS) were purchased from Sigma-Aldrich. Ethanol (EtOH, 99.8%), methanol (MetOH, 99.8%), and hydrochloric acid (HCl, 35–38%) were purchased from POCH Basic. Dulbecco’s Modified Eagle’s medium (DMEM), Hanks Balanced Salt solution, fetal bovine serum (FBS), Trypsine-EDTA (0.25%), penicillin-streptomycin, glutaraldehyde solution, and agarose were purchased from Sigma-Aldrich and used as received. WST-1 Cell Proliferation Assay Kit was purchased from Clontech. LIVE/DEAD® Viability/Cytotoxicity Kit for mammalian cells was obtained from Thermo Fisher Scientific. Formaldehyde methanol free, osmium tetroxide solution, uranyl acetate, and Embed-It*™* Low Viscosity Epoxy Kit were purchased from Polysciences.

### Preparation of GQDs

GQDs were prepared via the hydrothermal method (Permatasari et al. 2016; Qu et al. 2014), with the minor modification, using NaCitr (instead of citric acid) as a carbon precursor and TU as a base. NaCitr and TU reagents were mixed in the molar ratio of 1:3 (1.3 g:1.15 g), respectively. The reaction was run in water-based (25 ml) solution of both reagents in Teflon-lined stainless autoclave at 180 °C for 8 h. The formation of GQDs was indicated by the color change of the reaction solution to the orange one. Obtained postreaction solution was then given to vacuum drying at 60 °C and pressure of 200 mbar in order to remove the excess water. The final product was precipitated by adding EtOH to the solution and collected by centrifugation (13.2 rpm, 15 min), and dried at 60 °C. The obtained solid material can be easily dispersed in water.

### Preparation of MSNs

MSNs, as a carrier for GQDs, were synthesized using the previously reported template-directed sol-gel method, with the minor modifications (Nooney et al. 2002). In a typical used synthesis procedure 0.5 g CTAB, as the structure-directing agent, was dispersed in 240 ml of deionized water (H_2_O_d_) in a closed vessel under vigorous stirring at room temperature. After the solution became homogenous, its pH was adjusted to 11 with the 1 M (molar) NaOH solution and heated up to 80 °C. Subsequently, 2.5 ml of TEOS as the silicon source was dropwise added and the reaction solution was further stirred continuously for 3 h, until white precipitated was obtained. As-obtained product was collected by centrifugation (24,000 rpm, 15 min) and washing with MetOH and then dried overnight at 60 °C. In order to remove CTAB template, the final dried product was refluxed for 24 h in a mixture of 160 ml MetOH and 9 ml HCl (~37%). The obtained MSNs were centrifuged and washed with MetOH and H_2_O_d_, and finally dried at 60 °C.

### Preparation GQDs-MSNs nanocomposite nanoparticles

Prior to the GQDs-MSNs nanocomposites preparation, as-prepared MSNs were firstly amino-functionalized. For this purpose, 0.04 g MSNs was dispersed in EtOH and then treated with 0.8 ml APTES under the stirring. After 24 h, the suspension was centrifuged (13.2 rpm, 15 min) and washed with EtOH repeatedly for three times, in order to remove unreacted APTES. The prepared GQDs were then covalently immobilized onto amino-functionalized MSNs (NH_2_-MSNs) with the use of carbodiimide crosslinker chemistry. The amino-functionalized MSNs were dispersed in PBS-based solution (pH = 5.8) of GQDs (1 mg/ml) and added with 0.2 ml of EDC linker solution (20 mg/ml). After 24 h, the unbound QDs were removed by successive centrifugation and washing with EtOH and H_2_O_d_.

### DOX-loading and release studies

Doxorubicin HCl (DOX, Carbosynth Limited), a model chemotherapy drug, was non-covalently loaded onto prepared GQDs-MSNs nanocomposite nanoparticles, as well as onto un-modified MSNs. Briefly, suspension of nanocarrier was mixed with DOX solution (both prepared in PBS pH = 7.4, in a weight ratio of 2:1, respectively). A resulting reaction solution was given to continuous stirring for 24 h at the room temperature and in the dark. An obtained red-purple precipitate was separated by centrifugation, washed with PBS several times, and dried in air at 60 °C. The efficiency of DOX loading was determined by finding the difference in the DOX concentration in the solution before and after loading. DOX concentration was measured spectrophotometrically at 485 nm. Percentage of the loaded drug was calculated according to the following equation:

1 $$ \%{\mathrm{DOX}}_{\mathrm{loaded}}=\left[\left({\mathrm{C}}_{\mathrm{DOX}\ 0}\hbox{--} {\mathrm{C}}_{\mathrm{DOX}\ \mathrm{F}}\right)/{\mathrm{C}}_{\mathrm{DOX}\ 0}\right]\ast 100 $$

where *C*_DOX 0_ and *C*_DOX F_ are the initial and final DOX concentration in the reaction mixture.

To investigate drug release behavior from prepared GQDs-MSNs nanocomposites, as well as MSNs, 0.5 mg DOX-loaded nanocarrier was dispersed in 2 ml of PBS with pH 4.5, 5.0, 6.5, and 7.4 and given to continuous shaking under dark conditions. The temperature of PBS solution was kept constant 37 or 50 °C. After predetermined time intervals, the samples were centrifuged, then supernatant samples were withdrawn and replaced with fresh PBS for continuous drug release. The amount of released DOX was determined spectrophotometrically at 485 nm.

### Physicochemical characterization

The morphology and size of prepared GQDs were investigated using the transmission electron microscopy (TEM) and atomic force microscopy (AFM). TEM images were recorded with a high-resolution and analytical HRTEM Jeol ARM 200F operating at 200 kV or TEM Jeol JEM-1400 operating at 120 kV. AFM images were recorded in PeakForce Tapping® mode with the Icon Bruker microscope. Silicon nitride ScanAsyst-Air probes were used during the experiment and a freshly cleaved mica as a substrate. The crystal structure was characterized by X-ray diffraction (XRD) using Empyrean (PANalytical) diffractometer with Cu Kα-filtered radiation (1.54 Å). The vibrational properties of prepared samples were analyzed via attenuated total reflection (ATR) technique with a Tensor 27 spectrometer (Bruker), equipped with a MCT detector and a horizontal triple reflection PIKE MIRacle™ ATR accessory. Raman spectra were collected using an in Via Renishaw Raman Microscopy system with a 633-nm He/Ne laser and 1800 g/mm grating. The laser light was focused on the sample with a × 50/0.75 microscope objective (LEICA). N_2_ adsorption-desorption isotherms were obtained on Sorptometr Quantachrome NOVA 1000e. The surface area (SSA) and pore volume have been determined based on low temperature N_2_ sorption and estimated applying Brunauer-Emmett-Teller (BET) method and Barrett-Joyner-Halenda algorithm. Zeta potential and particle size distribution of prepared GQDs were measured on Zetasizer Nano ZS (Malvern Instruments Ltd.) based on electrophoretic light scattering (ELS) and non-invasive light scattering method (NIBS), respectively. The chemical composition of prepared GQDs was investigated using X-ray photoelectron spectroscopy (XPS). XPS spectra were obtained with an Sphera II photoelectron energy analyzer (Scienta Omicron) with the monochromatized Al Kα X-ray source mounted inside the UHV system. The absorbance and fluorescence excitation and emission spectra were acquired on UV-Vis-NIR spectrophotometer (Perkin Elmer lambda 950) and spectrofluorometer (FluoroSENS Gilden Photonics). The fluorescent quantum yield (QY) of prepared GQDs solutions in water was determined by applying the following equation:

$$ {\mathrm{QY}}_{\mathrm{sample}}=\mathrm{Q}{\mathrm{Y}}_{\mathrm{st}}\left(\frac{I_{\mathrm{sample}}}{I_{\mathrm{st}}}\right)\left(\frac{n_{\mathrm{sample}}^2}{n_{\mathrm{st}}^2}\right)\left(\frac{A_{\mathrm{st}}}{A_{\mathrm{sample}}}\right) $$

where QY is the quantum yield, *I* is the measured integrated emission, *A* is the absorbance at the excitation wavelength, and *n* is refractive index of the solvent. As a standard, the quinine sulphate solution in 0.1 M sulfuric acid solution was used (QY = 54.6%).

### Biochemical characterization

#### Cell culture

Human cervical cancer cell line HeLa obtained from American Type Culture Collection (ATCC) and human fibroblast cell line MSU1.1 obtained from Prof. C. Kieda (CBM, CNRS, Orléans, France) were used for in vitro studies. Cells were cultured in a complete medium DMEM supplemented with 10% FBS, 100 units/ml penicillin, and 100 μg/ml streptomycin, and grown at 37 °C in humidified atmosphere containing 5% CO_2_.

#### In vitro cytotoxicity assays

In order to determinate the cytotoxicity of GQDs, MSNs, and GQDs-MSNs nanocomposite nanoparticles, as well as DOX-loaded nanocomposite nanoparticles, HeLa and MSU1.1 cells were treated with increasing concentration of nanoparticles (from 20 to 400 μg/ml) and incubated for 24 h at 37 °C under a 5% CO_2_ atmosphere. Cells without any treatment were used as a negative control. The effect of the GQDs on cell viability was determined by WST-1 assay according to manufacturer’s instructions. Briefly, 10 μl of WST-1 solution was added to each well of 96-well plate and further incubated. After 2 h, the absorbance was measured with a microplate reader (Anthos Zenyth 340rt) at 450 versus a 650 nm reference. The relative cell viability (%) was expressed as a percentage relative to the negative control. Data are reported as the average ± standard deviation (SD) of experiments performed in triplicate. The cytotoxicity analysis was also performed for chemical inhibitors of cellular uptake (see, Fig. S4 in ESM).

The effect of the nanoparticles on cell viability was also determined using Live/Dead assay kit and analyzed by InCell Analyzer apparatus (GE Healthcare). Briefly, cultured adherent cells in a 96-well plate, previously co-incubated for 24 h with nanoparticles solutions at increasing concentration mentioned before, were prepared. Cells non-treated and treated with 50 *v*/*v*% dimethyl sulfoxide were used as negative and positive control (DMSO), respectively. Cells were washed with PBS, then a Live/Dead Viability Kit (Life Technologies) composed of two fluorescent dyes, calcein-AM and ethidium homodimer (EthD-1), for the staining of live and dead cells were used. Thus, in live cells, green fluorescence derived from calcein was observed in cytoplasm, whereas EthD-1 enters dead cells and was observed as red fluorescence in nucleus. Three repetitions for each condition were carried out. The images were acquired from 20 fields from each well, and then statistically analyzed by InCell Developer Toolbox software. The total cell number was normalized to the non-treated control group. Data are expressed as the average ± standard deviation (SD) of three different experiments.

### Cell uptake of GQDs- and GQDs-MSNs-imaging studies

#### CLSM imaging

To investigate the GQDs and GQDs-MSNs nanocomposites’ ability to cell penetration and their intracellular distribution, as well as to monitor DOX delivery and release, confocal microscopy was performed on cancer cell line (HeLa). Briefly, cells were plated on a chambered Lab-Tek dish (1 × 10^4^ cells/well), grown overnight, and then were incubated at 37 °C with GQDs, GQDs-MSNs nanocomposites (20 μg/ml) for 3 or 24 h. The cells were then rinsed three times with PBS (pH 7.4). The distribution of GQDs, GQDs-MSNs nanocomposites and doxorubicin was analyzed using a confocal laser scanning microscope (CLSM, FV1000, Olympus). Colocalization of DOX with GDQ-MSN was analyzed via Pearson’s coefficient constant.

#### Tracking pathway of cellular uptake

In order to block energy-dependent mechanisms of GQDs-MSNs uptake, the grown HeLa cells were incubated at 4 °C for 1 h. Media was then replaced with cold serum-free DMEM containing 20 μg/ml of GQDs-MSNs and incubated for another 3 h at 4 °C. Afterward, cells were rinsed with PBS, maintained in phenol red-free medium, and imaged using a laser scanning confocal microscope (CLSM, Olympus FV1000).

The influence of different endocytic inhibitors on the cellular uptake of GQDs-MSNs was also assessed. Briefly, the seeded HeLa cells were incubated separately with (1) methyl-β-cyclodextrin (2,5 mg/ml), as an inhibitor of caveolae/lipid raft-dependent endocytosis; (2) chlorpromazine hydrochloride (5 μg/mL), as an inhibitor of clathrin-mediated endocytosis; and (3) wortmannin (150 ng/mL), as macropinocytosis inhibitor, for 1 h at 37 °C. Subsequently, cells were incubated with 20 μg/ml of GQDs-MSNs nanoparticles nanocomposite for 3 h and imaged as mentioned above. The concentration of different inhibitors were chosen based on performed earlier their cytotoxicity analysis (Fig. S2 in ESM).

#### TEM imaging

The cellular uptake and distribution of GQDs-MSNs nanocomposites in cells was further analyzed by TEM (Jeol JEM-1400) according to Graham and Orenstein (Graham and Orenstein 2007) procedure with minor modification. Briefly, HeLa cells previously incubated with 100 μg/ml of GQDs or GQDs/MSNs nanocomposites for 4 h were fixed with 2.5% glutaraldehyde solution for 2 h and then postfixed in 1% osmium tetroxide for 1 h at room temperature. Then, samples were dehydrated through a graded series of ethanol concentrations (50, 70, 80, 90, 96, and 100%) and embedded in epoxy resin. Ultrathin sections were prepared using ultramicrotome (RMC PowerTome PT-XL), collected onto TEM grids, stained with 1% uranyl acetate, and imaged under Jeol JEM-1400 TEM.

## Results and discussion

### Preparation and characterization of GQDs and GQDs-MSNs

For the synthesis of GQDs, the bottom-up approach was chosen, as it is reported to yield better quality GQDs concerning their morphology, size distribution, and optical properties (Bacon et al. 2014). After the hydrothermal reaction, a bluish solution with blue emission under UV light was obtained. From the HRTEM images (Fig. S1a,b in ESM), a clear interplanar distance of about 0.35 nm can be found as marked particularly in the Fig. S1b, which corresponds to that of the (002) d-spacing of graphite (Qu et al. 2013) confirming that prepared GQDs have a graphite-like nature. As presented in the HRTEM images, the crystal structure of GQDs is strongly defected; hence, it is difficult to indicate accurate hexagonal ordering typical for graphene. Prepared GQDs are uniform in size (ranging from 1.5 to 7.6 nm), with an average particles diameter of 3.65 nm (Fig. S1c in ESM). AFM images (Fig. S1 e, f in ESM) confirm that uniform GQDs were prepared, as they show a topographic height of nanostructures up to 1.15 nm. Raman spectrum of prepared GQDs reveals prominent D, G, D’, and 2D bands at 1328.6 cm^−1^, 1575.8 cm^−1^, 1611.2 cm^−1^, and 2642.6 cm^−1^, respectively. G band is a result of in-plane vibrations of sp^2^ bonded carbon atoms, whereas D and D’ bands are due to the out of plane vibrations attributed to the presence of structural defects. Found *I*_*D*_/*I*_*G*_ ratio is around 0.86, which indicates that sp^2^ bonds of the carbon are disrupted by the presence of oxygen-containing functional groups (originating from the precursor), and therefore prepared GQDs are considered as highly defected materials.

The XPS elemental analysis of prepared GQDs reveals the presence of carbon, oxygen, nitrogen, sulfur, and sodium. The survey and relevant core level C 1s, O 1 , N 1s spectra and S 2s, S 2p region spectra are given in Fig. S2 in ESM. C 1s core level spectrum is composed of three peaks at around 284.5 eV, 286.0 eV, and 288.2 eV that can be assigned to the sp^2^ C in graphene (C-C, C=C bonds), sp^3^ C in C-O, C-N bonds and O-C=O, respectively (Qu et al. 2013). The O 1s core level peaks at around 531.3 and 532.6 eV correspond to oxygen in states of C=O, C-O-C/C-OH, respectively (Qu et al. 2013). As shown, C=O-related oxygen states are dominating. The C=O groups are formed by the intramolecular dehydrogenation of carbon precursor, and the other C-O-C/C-OH are remaining surface and edge-related epoxy groups and carboxylic functional groups, resulting from the incomplete dehydrogenation and carbonization during the synthesis. Moreover, N 1s core level spanning from around 396.0 to 403.0 eV is clearly resolved, as well as S 2s and S 2p core levels at around 227.0 and 163.0 eV, respectively (Qu et al. 2013). Elemental XPS analysis reveals that prepared GQDs contain 72.34 at% of C, which is a high content in comparison to other results (Hao et al. 2015), 13.97 at% of O, 4.97 at% of N and 5.39 at% of S, hence confirming N and S-doping of GQDs.

These results confirm that obtained GQDs are rich in carbon, doped with N and S atoms, but still contain number of oxygen-containing functional groups and exhibit disordered and disrupted structure, what is likely for zero-dimensional (0D) nanostructures in comparison to two-dimensional (2-D) graphene nanosheets (Kozak et al. 2016).

**Fig. 1:**
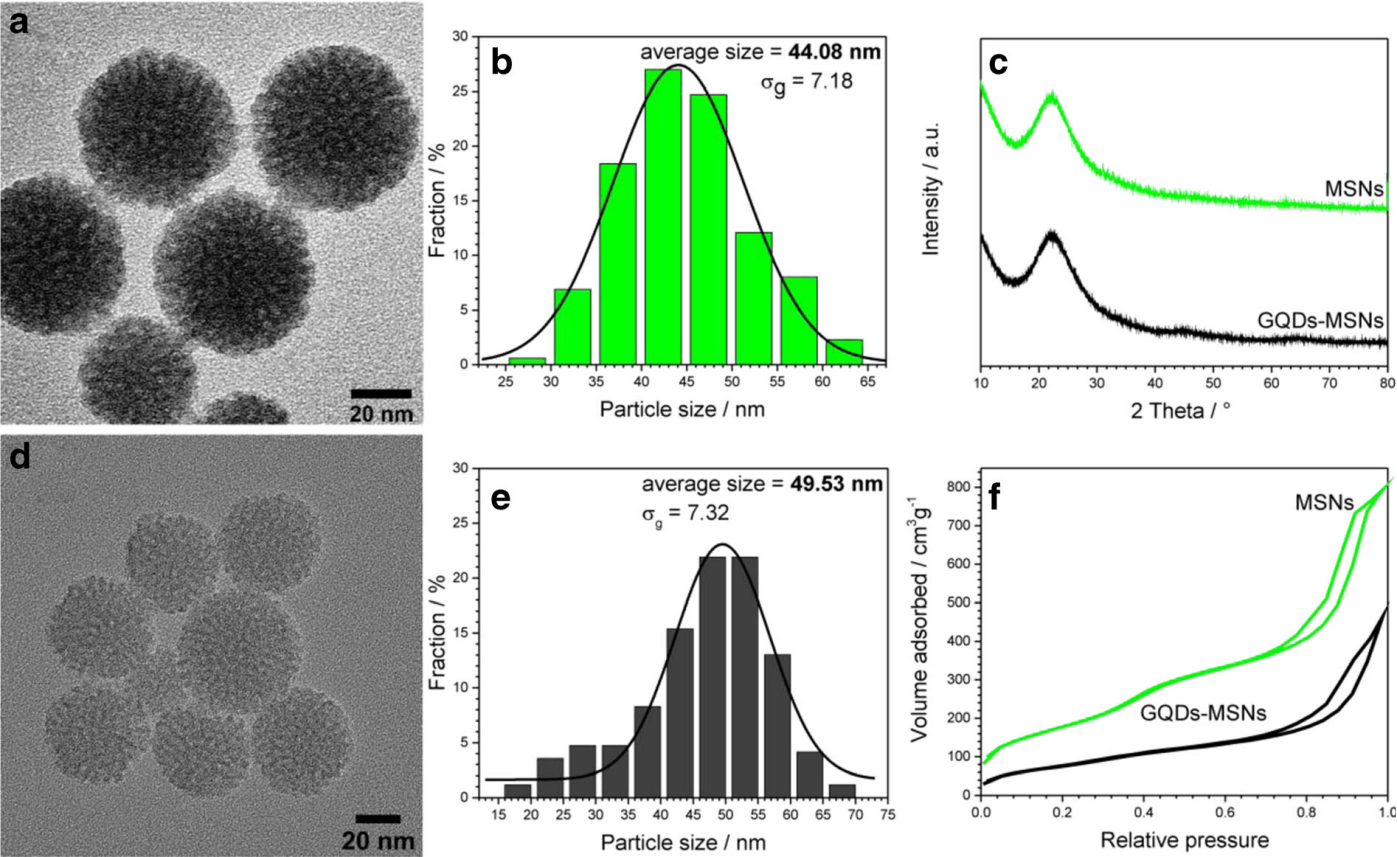
TEM images and particle size distribution of prepared MSNs (**a**, **b**) and GQDs-MSNs nanocomposite nanoparticles (**d**, **e**); XRD patterns (**c**) and N_2_ adsorption/desorption isotherms (**f**)

During the synthesis of MSNs, the pH and temperature were carefully controlled in order to obtain nanostructures with size below 50 nm, as their eventual biomedical application is foreseen. As shown in the TEM images (Fig. 1a), obtained silica nanoparticles are spherical and dendritic structures, with the average particle size of 44.08 nm and quite uniform size distribution (Fig. 1b). Their average particle size slightly increased up to 49.53 nm upon GQDs immobilization (Fig. 1d, e). N_2_ adsorption-desorption isotherms (Fig. 1f) also confirm that mesoporous silica nanoparticles were obtained, as they can be characterized with type IV isotherm, exhibiting a hysteresis loop. After immobilizing the GQDs onto MSNs, obtained GQDs-MSNs maintained mesoporous structure; however, the N_2_ adsorption amount decreased sharply. Similarly, upon GQDs immobilization, the surface area (SSA) and pore volume decreased from 442.33 to 197.40 m^2^/g and from 1.06 to 0.65 cm^3^/g. These results confirm indirectly that GQDs were successfully immobilized onto MSNs. The broad diffraction peak at 15–30 2*θ*° indicates that prepared MSNs have an amorphous structure (Fig. 1c). The XRD pattern does not reveal any structural changes of MSNs upon GQDs immobilization. The mesoporous structure with high surface area and pore volume indicate that prepared GQDs-MSNs have a great potential for drug-loading efficiency.

**Fig. 2:**
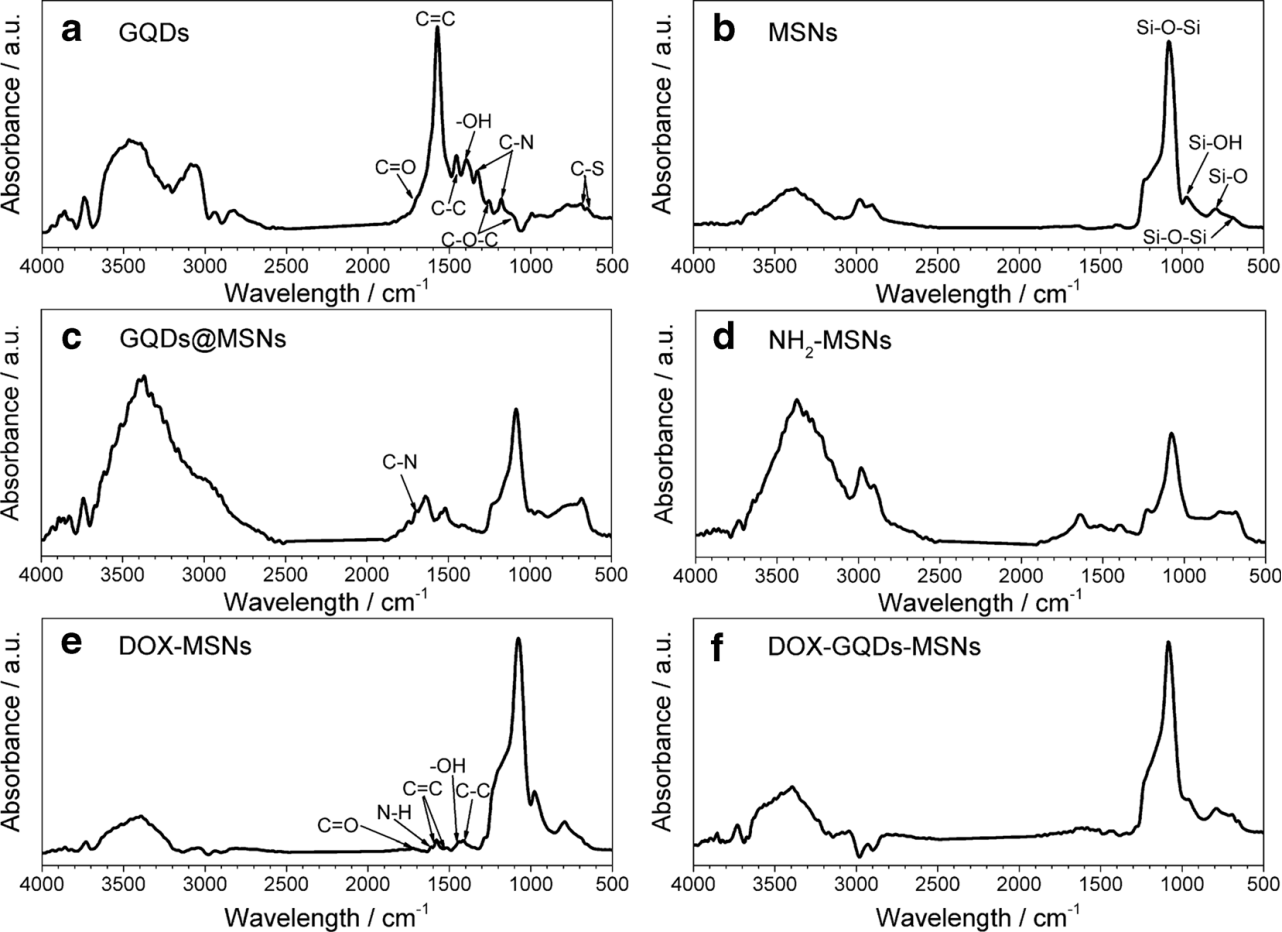
FTIR spectra of prepared: GQDs (**a**), MSNs (**b**), GQDs-MSNs (**c**), amino-functionalized NH_2_-MSNs (**d**), and loaded with doxorubicin DOX-MSNs (**e**) and DOX-GQDs-MSNs (**f**)

The successful preparation of GQDs, MSNs, and GQDs-MSNs nanocomposite nanoparticles, as well as their successive modifications and loading with DOX are revealed and confirmed with the FTIR spectroscopy (Fig. 2). FTIR spectrum of GQDs (Fig. 2a) is featured IR absorption bands typical for graphene derived structures, namely (i) C-O-C stretching (1109 and 1254 cm^−1^); (ii) phenolic -OH stretching (1395 cm^−1^); (iii) C-C (1457 cm^−1^); (iv) aromatic C=C (1572 cm^−1^), but also C=O stretching (1726 cm^−1^) of carboxylic groups (Yao et al. 2017a, b; Yao et al. 2017a, b). In the 2800–3000 cm^−1^ region, the C-H-related bands are exhibited: aldehyde doublet, methylene asymmetric stretching, and methyl asymmetric stretching at 2829 cm^−1^, 2845 cm^−1^, and 2940 cm^−1^, respectively. In turn, bands observed in the range of 3000–4000 cm^−1^ could be attributed to hydroxyl and amine groups. The presence of -NH groups is strictly related to remaining impurities of thiourea applied during the synthesis process. This could be additionally confirmed by the presence of C-N at 1329 and 1179 cm^−1^, as well as C-S at 690- and 650 cm^−1^-stretching vibrations. Therefore, these IR spectra confirm that hydrothermally prepared GQDs are rich in oxygen-containing functional groups, thus have a potential to react further with NH_2_-MSNs. In case of MSNs-based samples (Fig. 2b–f), all the spectra are featured by presence of Si-O-Si bending vibration, Si-O symmetric stretching, Si-OH symmetric stretching, and Si-O-Si asymmetric stretching in range of 680–695 cm^−1^, 780–795 cm^−1^, 945–990 cm^−1^, and 1075–1090 cm^−1^, respectively. Moreover, one can notice scissor-bending vibrations of adsorbed molecular water in range of 1615–1645 cm^−1^, as well as H-bonded silanol -OH groups in range of 3365–3395 cm^−1^ and 3635–3650 cm^−1^. From the spectrum of bare MSNs (Fig. 2b), one can notice the presence of asymmetric -CH_2_ and -CH_3_ stretching vibrations at 2904 and 2979 cm^−1^, respectively. The presence of methylene and methyl groups could be related to remaining impurities like unreacted Si-precursor TEOS or surfactant CTAB, used during the synthesis. In case of NH_2_-MSNs sample (Fig. 2d), one can observe intensity decrease of the Si-O symmetric stretching vibration along with the disappearance of Si-OH symmetric stretching, which proves successful MSNs functionalization with aminosilane compound. However, the amine groups vibrations are overlapped by free or adsorbed -OH groups. Therefore, -NH_2_ presence is confirmed by the broadening of the band in range of 3050–3700 cm^−1^. Introduced amine groups were further used to covalently bind GQD to MSNs via amide bond (C-N), which is present in GQD@MSNs spectrum at 1695 cm^−1^ (Fig. 2c), next to the bands typical for MSNs and GQD previously described. Finally, the adsorption of the doxorubicin onto MSNs was also investigated. Figure 2e shows the IR spectrum of DOX-MSNs, where the presence of drug is confirmed with the following bands: C-C stretching (1413 cm^−1^), -OH bending (1444 cm^−1^), aromatic C=C vibrations (1516 and 1580 cm^−1^), N-H bending (1611 cm^−1^), as well as C=O stretching (1726 cm^−1^) of carboxylic groups. All of the doxorubicin-related bands can be also observed in the spectrum of DOX-GQDs-MSNs (Fig. 2f). The positions of the peaks are slightly up-shifted (+ 5 cm^−1^), and their intensities are decreased. Moreover, the contribution of typical bands for graphene structures diminishes upon DOX loading, which indicates the stronger interaction between GQDs and DOX molecules. Nevertheless, these results confirm successful formation of designed drug delivery system.

Due to the foreseen biomedical application of prepared GQDs-MSNs nanocomposite nanoparticles, they should form stable suspensions in water-based media. Therefore, the zeta potential was controlled at each stage of their preparation, as well as after surface functionalization and further doxorubicin drug attachment. The results are given in Table 1. Both prepared bare GQDs and MSNs exhibit moderately high zeta potential in water-based suspension and are negatively charged, − 24.2 and − 30.0 mV, respectively. This is in agreement with reported isoelectric point and zeta potential of MSNs (Wu et al. 2013a) and of GQDs (Wu et al. 2013b). The negative charge is resultant of number of oxygen-containing functional groups at both GQDs and MSNs, in agreement with FTIR spectroscopy. After modifying with amino groups, the zeta potential of NH_2_-MSNs changed to the positive one with higher value of + 34.1 mV. In turn, after covalent immobilization of GQDs, the zeta potential remained positive and exhibited value of + 31.1 mV for GQDs-MSNs. Hence, these results confirm successful functionalization and preparation of GQDs-MSNs nanocomposite nanoparticles. Their high zeta potential is an advantage, as they are considered to form relatively stable suspensions. Moreover, the positive charge of GQDs-MSNs will enable their efficient cellular uptake via the electrostatic interaction with negatively charged cell membranes (Frohlich 2012).

**Table 1:** Summary of the physicochemical properties: the average particle size (*d*_TEM_) and standard geometric deviation (*σ*_*g*_), the surface area (SSA) and pore volume (V), zeta potential (ζ) results measured in water-based (H_2_O_d_) suspensions; DOX-loading and release efficiency (% DOX_loaded_, loading capacity in μg DOX/1 mg NPs, and release in %)

Sample name	*d*_(TEM)_/*σ*_*g*_	SSA (cm^2^/g)/V (cm^3^/g)	Zeta potential (mV)/std. dev.	
MSNs	44.08/7.18	442.33 / 1.06	−30.0 / 0.3	
NH_2_-MSNs	–	–	+34.1 / 0.7	
GQDs	3.65/0.81	–	−24.2 / 1.2	
GQDs-MSNs	49.53/7.32	197.40 / 0.65	+31.1 / 0.8	
DOX-MSNs	–	–	−6.9 / 1.6	
DOX-GQDs-MSNs	–	–	+30.8 / 1.8	
Sample name	% DOX_loaded_	Loading capacity (μg DOX/1 mg NPs)	Release at pH 5.0 after 48 h (%)	
			37 °C	50 °C
DOX-MSNs	78.88	399.4	55.33	89.12
DOX-GQDs-MSNs	53.93	269.7	26.96	28.42

### Optical properties

**Fig. 3:**
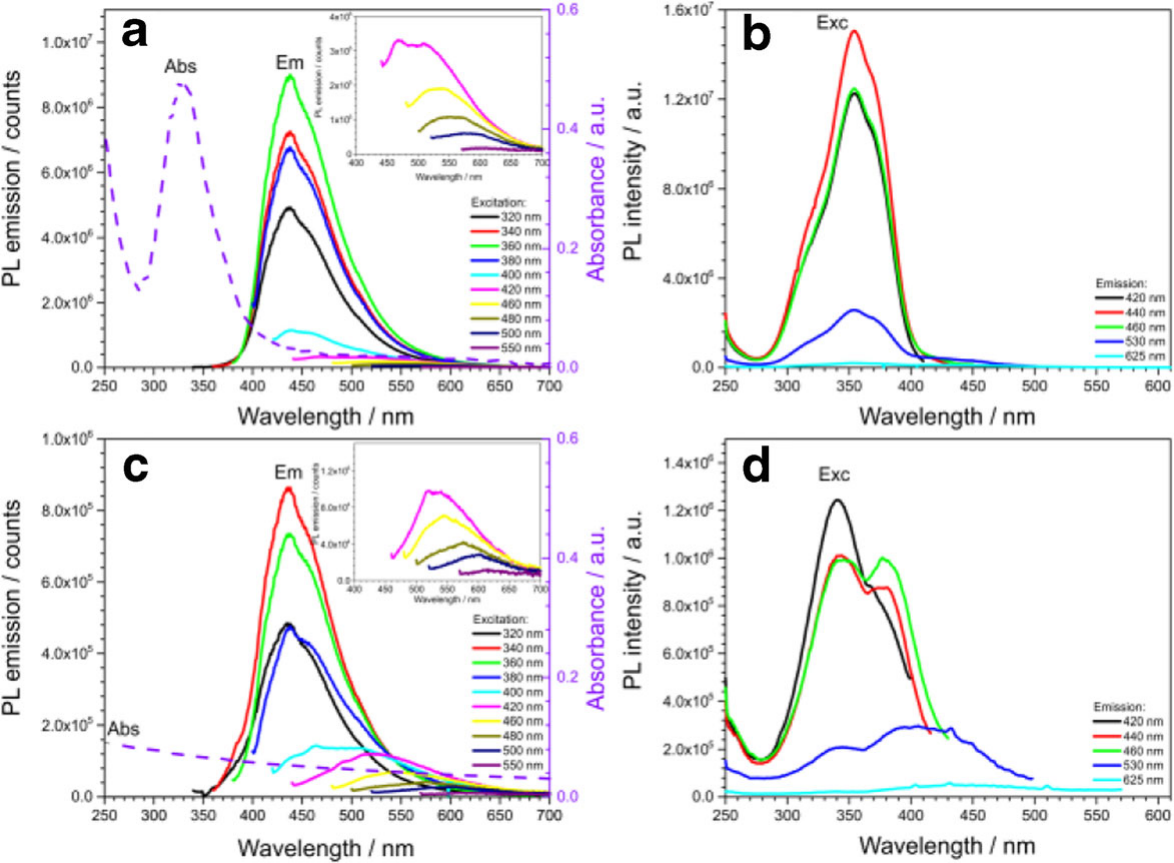
Optical properties of prepared GQDs (**a**, **b**) and GQDs-MSNs nanocomposite nanoparticles (**c**, **d**): excitation-dependent PL emission (Em) spectra and absorbance (Abs) spectra (**a**, **c**); PL excitation (Exc) spectra (**b**, **d**)

Surface passivation with carbonyl groups and heteroatom doping, revealed by FTIR and XPS, find the reflection in the optical properties, as they change the electronic density of bulk semiconductor materials (Zhu et al. 2012; Permatasari et al. 2016). The UV-Vis absorption and photoluminescent (PL) excitation and emission properties were investigated using water-based suspensions of both GQDs and GQDs-MSNs (Fig. 3). The PL quantum yield of GQDs for the emission at 440 nm was calculated to be 39.5%. In the UV-Vis absorbance spectra of GQDs, a strong absorption peak at 330 nm is observed, which corresponds to the n-π* transition of the conjugated C=O bonds in carbonyl groups present at the surface and edges of prepared GQDs and of possible C=N bonds (Qu et al. 2014), the presence of which was revealed by XPS and FTIR. By the contrast, the prepared GQDs-MSNs suspension reveal high absorbance background of decreasing intensity with increasing wavelength. The PL excitation spectra (Fig. 3b, d) are consistent with the absorbance spectra, as their maxima overlap with the absorbance maxima. The highest PL emission intensity is obtained for the excitation between 340 and 360 nm (Fig. 3a, c). GQDs and GQDs-MSNs solutions exhibit the blue PL emission with the maximum peak located at around 440 nm under UV excitation from 320 to 360 nm. GQDs-MSNs solution reveals significantly lower PL emission intensity. Maximum intensity of PL emission is observed under excitation wavelength of 360 and 340 nm for GQDs and GQDs-MSNs, respectively. The Stokes shift is therefore about 80 nm for GQDs and 95 nm for GQDs-MSNs. Under excitation above 400 to 550 nm the maximum PL emission peak shifts towards longer wavelengths from 440 to about 600 nm, along with the intensity decrease. Therefore, the PL emission of prepared GQDs and GQDs-MSNs can be considered as excitation-dependent, with three main PL regions (blue emission for the excitation up to 400 nm, green emission for the excitation below 480 nm, and red emission for the excitation below up to 550 nm). This effect will be particularly presented in the bioimaging studies. The excitation-dependent PL emission has been previously observed in GQDs, and it was related to the presence of GQDs with different sizes or conjugated sp^2^ domains (Sk et al. 2014). Therefore, we can conclude that the blue emission results from the chromophores related to C=O bonds (observed in absorbance spectrum). The green and red emission therefore must have an another chromophore origin, possibly related to the C=N bonds (Qu et al. 2014), but also due to the presence of different sized GQDs (Sk et al. 2014). This is also well reflected in the PL excitation spectra for the emission above 420 nm, which reveal more than one excitation maximum, dominating one at 330 nm (as in absorbance spectra), but also excitation peaks about 370 nm, in the dominating peak shoulder. This second excitation contribution is more pronounced (increasing for the emissions at longer wavelengths) in case of GQDs-MSNs. This suggests the scenario of forming clusters of GQDs at the MSNs surface, which retain the fundamental optical properties of the individual GQDs, however with an influence on their PL emission intensity in the water-based solutions. Effect of the clustering of QDs on their optical properties and resulting application as bioimaging labels has been investigated, e.g., for CdSe/CdS quantum dots and quantum rods (Rafipoor et al. 2015) or more recently in ZnAgInSe/ZnS QDs (Deng et al. 2017).

### DOX-loading and release profile

To study the anticancer drug-loading and release efficiency, doxorubicin hydrochloride was non-covalently loaded onto prepared GQDs-MSNs nanocomposite nanoparticles, as well as onto un-modified MSNs as a control drug carrier. The advantage of the adsorption interaction due to the high surface area of mesoporous carrier, as well as electrostatic attraction was exploited for this purpose. The DOX-loading efficiency (% DOX_loaded_) was estimated spectrophotometrically to be 53.93 (which corresponds to the loading capacity of 269.7 μg/mg) and 78.88% (which corresponds to the loading capacity of 399.4 μg/mg) for GQDs-MSNs and MSNs, respectively. As expected, the drug-loading efficiency was higher in case of control un-modified MSNs, what can be assigned to their high surface area and pore volume, but also to the efficient electrostatic attraction between positively charged DOX molecules and negatively charged MSNs. When considering DOX-loading onto GQDs-MSNs, the drug-loading efficiency was reduced, firstly by their lower specific surface area and pore volume, which were blocked by the GQDs immobilized onto MSNs. Secondly, the drug-loading efficiency through the electrostatic attraction is unfavorable to take place owing to the positive charge of GQDs-MSNs. Results of the zeta potential measurements (see, Table 1) show that due to the DOX-loading the electrophoretic potential of DOX-GQD@MSN suspension does not change significantly and remain positive (+ 30.8 mV). Whereas for DOX-MSNs, the significant zeta potential decrease is observed; however, it remains negative value (− 6.9 mV). These results indicate that water-based suspensions of prepared DOX-GQDs-MSNs exhibit better stability than DOX-MSNs; hence, these nanocomposite nanoparticles can be considered as more preferred carrier for drug delivery application.

**Fig. 4:**
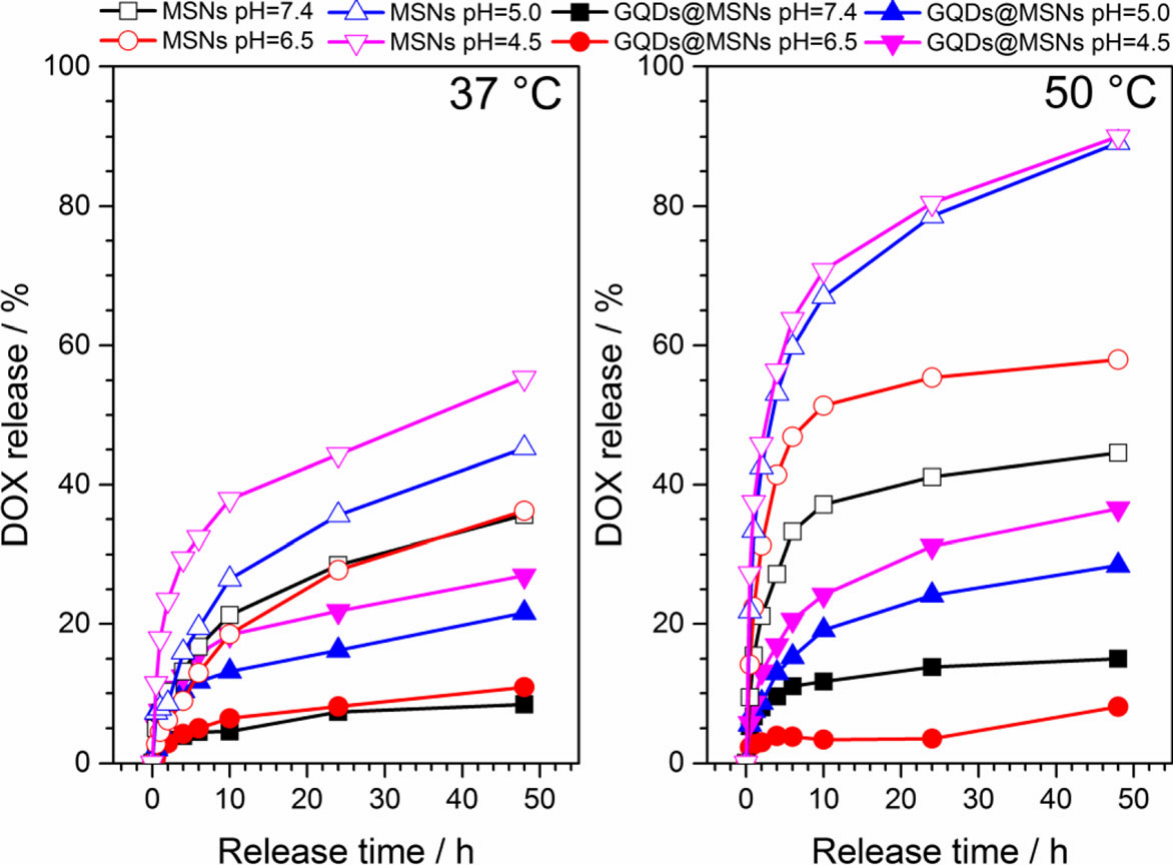
The release of doxorubicin (DOX) from MSNs and GQDs-MSNs at different temperatures (37 and 50 °C) and different pH values

The drug release behavior was investigated in response to different pH (4.5, 5.0, 6.5, 7.4) and temperature (37 and 50 °C) in PBS-based release solution. The acidic and neutral pHs of a release solution were chosen as they reflect best the tumor environment (endosomes and lysosomes of cancer cells) and a physiological environment in normal tissue, respectively (Upreti et al. 2013). Figure 4 shows the cumulative DOX release from DOX-GQDs-MSNs and DOX-MSNs under different conditions. All profiles exhibit gradual release over the time, without the rapid initial DOX release. The release of DOX was fastest in the medium with pH 4.5 and 5.0 and at 50 °C for both prepared carriers. Obviously, the release of DOX was more efficient in case of DOX-MSNs, and 90.02 and 89.12% of loaded drug was released within 48 h at 50 °C, respectively at pH 4.5 and 5.0. By contrast, DOX-GQDs-MSNs exhibited slower release rate, and 36.55 and 28.42% of loaded drug was released within 48 h at 50 °C, respectively at pH 4.5 and 5.0. This can be ascribed to the lower drug-loading efficiency, but also to the stronger type of interaction between DOX molecules and GQDs-MSNs, as shown with FTIR analysis, in comparison to the weak adsorption and electrostatic attraction in case of DOX-MSNs. Therefore, also the temperature effect was more significant in case of DOX-MSNs, where the temperature increase could weaken the interaction and facilitates DOX molecules release. It can be also concluded that both DOX-GQDs-MSNs and DOX-MSNs carrier exhibit a pH-dependent drug release behavior, as the DOX release can be triggered by acidic conditions, due to the weakening of the interaction between DOX molecules and GQDs-MSNs and MSNs. Hence, the drug delivery and release in cancer cells could be enhanced by the use of proposed nanocarriers, and further could result in diminishing the side effects of DOX. Although the low DOX-release efficiency is observed for DOX-GQDs-MSNs, the released amount of drug is enough to trigger the cytotoxic effects and what will be further presented.

### In vitro cytotoxicity

**Fig. 5:**
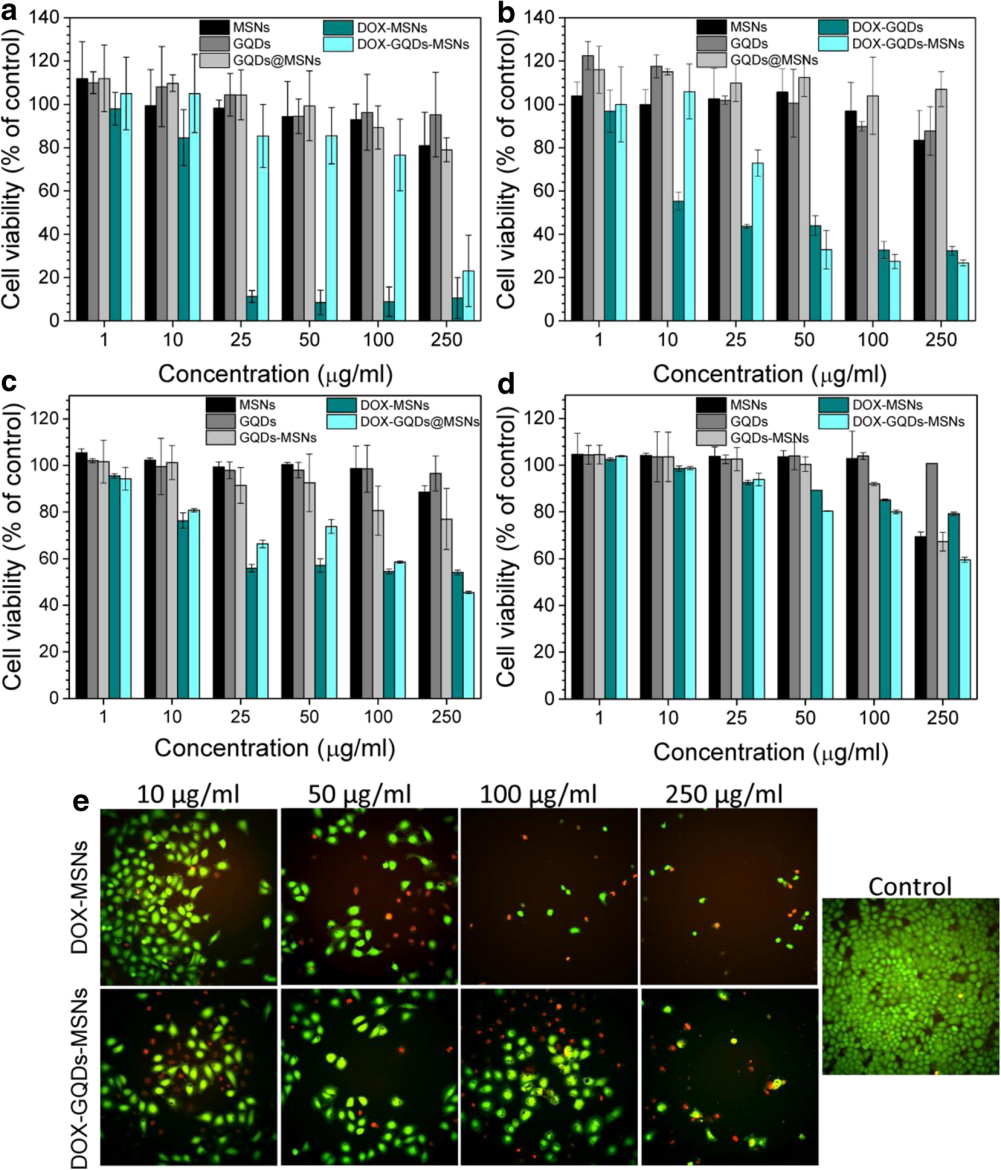
Cell viability of HeLa cells (**a**, **c**) and MSU1.1 fibroblasts (**b**, **d**) after 24 h of incubation with different concentration of MSNs, GQDs, GQDs-MSNs, and loaded with drug DOX-MSNs and DOX-GQDs-MSNs, investigated with WST-1 assay (**a**, **b**) and Live/Dead assay kit and analyzed by InCell Analyzer apparatus; representative fluorescence images showing the treatment efficacy of DOX-MSNs and DOX-GQDs-MSNs on the HeLa cells viability and proliferation using calcein-AM and EthD-1 fluorescence staining (**e**). Cells with green fluorescence are considered as viable cells, whereas those with red fluorescence are considered as dead cells

In order to estimate the cytotoxicity of GQDs, MSNs as well as GQDs-MSNs nanocomposite nanoparticles, the WST-1 assay was firstly performed. This colorimetric assay is based on the cleavage of a tetrazolium salt by mitochondrial dehydrogenases forming formazan in viable cells. Therefore, the greater the amount of formazan is produced following the addition of WST-1, the greater the number of metabolically active viable cells. Figure 5a, b shows the cell viability of cervical cancer cells (HeLa) and normal human fibroblasts (MSU1.1) after incubation for 24 h with a series of concentrations of GQDs, MSNs, and GQDs-MSNs, also loaded with doxorubicin. The results show that GQDs, MSNs, and GQDs-MSNs only slightly affect the viability of both cells type, which remained above 90%. Only the highest concentration (250 μg/ml) of nanoparticles was more deleterious to cells; however, even in this case, the viability remained above 80%.

The loading of doxorubicin onto MSNs or GQDs-MSNs generally results in a decrease of cells viability in a concentration-dependent manner. In case of HeLa cells, the decrease after incubation with DOX-MSNs proceeds very rapidly from the 25 μg/ml nanoparticles concentration, and more than 80% of cells are indicated as death. Whereas, for DOX-GQDs-MSNs-treated cells a marked decrease in viability is observed at higher concentration of 250 μg/ml. This is in agreement with the presented above DOX-release profile, showing that the amount of released DOX from DOX-GQDs-MSNs is much lower than from DOX-MSNs, therefore justifying the need for higher nanocarrier concentration in this case, but not a higher dose of DOX itself.

In comparison, for human fibroblasts, the viability decrease is milder; however, for DOX-MSNs-treated cells, the 50% inhibition of the cell viability is observed already at the concentration of 10 μg/ml and then decline gradually. The cytotoxicity of DOX-GQDs-MSNs initially seems to be lower, but ultimately at higher concentrations (from 50 μg/ml) reaches higher value than for DOX-MSNs.

The WST-1 results on the cytotoxicity of prepared nanomaterials, reflecting the metabolic activity of cells, were then compared to plasma membrane integrity of cells, indicated by the intracellular esterase activity with the LIVE/DEAD assay (Fig. 5c, d). Here, two dyes were used, which quickly discriminates live from dead cells by simultaneous staining with green-fluorescent calcein-AM, indicating intracellular esterase activity and red-fluorescent ethidium homodimer-1, indicating a loss of plasma membrane integrity. In addition, the fluorescence images acquired with the InCell Apparatus (Fig. 5e) give an overview on the cell viability but also proliferation. In case of nanoparticles without doxorubicin, obtained results are comparable with those from WST-1 assay. The only difference is observed in case of fibroblasts, where at the highest concentration of MSNs and GQDs-MSNs the viability does not exceed 70%. Moreover, evaluation of DOX-MSNs and DOX-GQDs-MSNs cytotoxicity based on the cell membrane integrity analysis shows that these drug carriers are not as cells deleterious as it was presented above with the metabolic activity analysis. It is presented (particularly for fibroblasts), that even at the highest applied doses of 250 μg/ml, the viability was maintained at 80 and 60% for DOX-MSNs and DOX-GQDs-MSNs, respectively. However, as presented in Fig. 5e, the treatment with DOX-loaded nanoparticles has a strong effect on the cell proliferation, which is particularly the case of DOX-MSNs, in comparison to control non-treated cells. Therefore, found with these two assays differences in the cytotoxicity of prepared nanoparticles, arise from the fact that different aspects of cell functions were analyzed, cell viability and cell vitality, by live/dead assay and WST-1, respectively (Kwolek-Mirek and Zadrag-Tecza 2014). However, both are required for the estimation of the physiological state of a cell after exposure to various types of stressors, including nanoparticles. Whereas, the fluorescent staining distinguishes live from dead cells, the WST-1 assay gives the information about their ability to reproduction. Hence, the lower number of living cells upon DOX-MSNs and DOX-GQDs-MSNs indicates the action of successfully delivered and released doxorubicin. Further, both assays confirmed that conjugation of biocompatible GQDs with nontoxic MSNs, leads to the creation of low-cytotoxic GQDs-MSNs nanocomposite nanoparticles, making them particularly good candidates for drug-delivery system.

### Intracellular localization and uptake mechanism of GQDs-MSNs

**Fig. 6:**
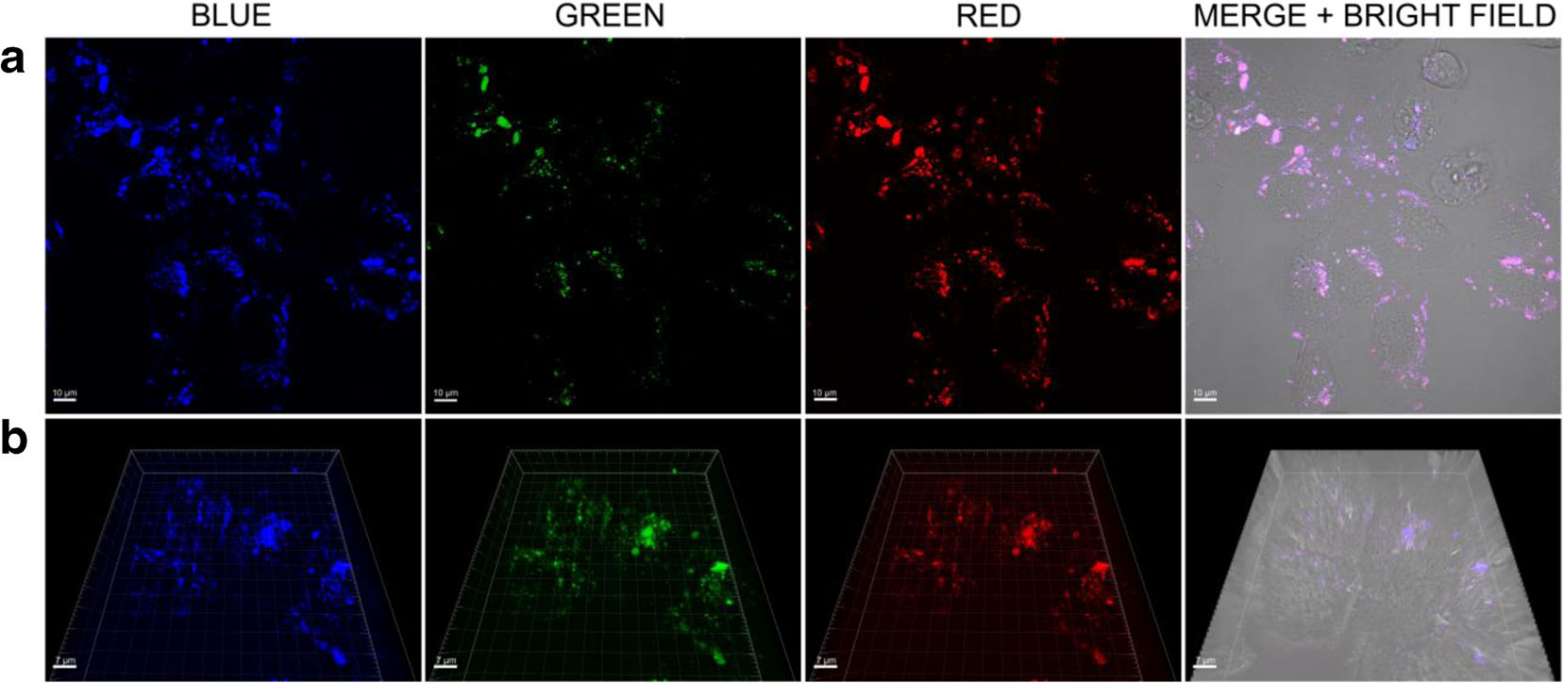
2D (**a**) and 3D (**b**) CLSM imaging and intracellular localization of GQDs-MSNs in HeLa cells after 4 h of incubation (50 μg/ml). Channels blue, green, and red represent the fluorescence of GQDs in GQDs-MSNs at the excitation 405 nm, 488 nm, and 559 nm, respectively (laser power of 0.5%)

The use of GQDs with their PL properties allows to monitor cellular uptake and distribution of GQDs-MSNs nanocomposite nanoparticles directly. Moreover, the GQDs entrapment onto mesoporous silica nanoparticles structure resulted in the increasing fluorescent signal, especially in the red channel in comparison to bare GQDs (Fig. S3 in ESM). The intensification of obtained fluorescence signal could be related with immobilization and aggregation of GQDs onto MSNs, of what was previously suggested (Rafipoor et al. 2015; Deng et al. 2017), but also to their more effective penetration into cells, enabled by their preferred positive surface charge for the interaction with negatively charged cell membrane (Frohlich 2012). To demonstrate the fluorescence imaging performance of GQDs-MSNs nanocomposite nanoparticles, the in vitro cellular uptake experiments were performed using HeLa cells. Cells were exposed to 50 μg/ml of nanocomposite nanoparticles for 4 h and afterwards analyzed by CLSM. The results are presented in Fig. 6. Here, the excitation-dependent PL behavior of the GQDs can be observed. Confocal images of HeLa cells incubated with nanoparticles upon 405 nm excitation exhibit the fluorescence emission in blue region, which could be observed as signals inside the cells. When the excitation light changes to 488 nm, green fluorescence is observed; finally, upon 559 excitation, the red fluorescence is emitted. A similar effect was observed by Zhu et al. (Zhu et al. 2011) for GQDs, which however emitted green and yellow fluorescence. According to the merged images, GQDs-MSNs after 4 h of co-incubation were internalized by HeLa cells and localized in the cytoplasm. The internalization was clearly confirmed in the 3D images (Fig. 6b). It has been already proved that GQDs can be easily internalized into cells (Markovic et al. 2012; Yuan et al. 2014; Schroeder et al. 2016). Likewise, Lu et al. (Lu et al. 2007) demonstrated that MSNs nanoparticles are readily endocytosed by cells and usually enter cells in an energy-dependent manner.

In order to evaluate the exact uptake mechanism of GQDs-MSNs, the inhibition of selected routes were performed. Results given in Fig. S5 (ESM) indicate that by lowering the temperature to 4 °C, the energy-dependent mechanisms is blocked. This is observed by lacking signal in cells, is comparison to control HeLa cells treated with 20 μg/ml GQDs-MSNs for 3 h at 37 °C, where the signal in cells is clear. Therefore, the endocytosis is considered as an exact uptake mechanism of studied nanoparticles. However, by using suitable inhibitors, different types of endocytosis were further investigated. Chlorpromazine is reported to specifically inhibit clathrin-dependent endocytosis (CDE), while methyl-β-cyclodextrin has been extensively used to inhibit clathrin-independent endocytosis (CIE), especially in caveolae/lipid raft-mediated endocytosis (Le et al. 2002; Parton and Richards 2003, dos Santos et al. 2011). Wortmannin, in turn, as covalent inhibitor of phosphoinositide 3-kinases (PI3Ks), plays a role in inhibition of micropinocytosis (Araki et al. 1996; Rupper et al. 2001). As can be seen in Fig. S5, chlorpromazine showed no inhibitory capacity in HeLa cells on studied nanoparticles uptake. Contrary to chlorpromazine, the MβCD inhibited the uptake of GQDs-MSNs into cells completely, suggesting the CIE as a main route of these nanoparticles internalization. Similar results were presented by Ekkapongpisit et al. (Ekkapongpisit et al. 2012), contrary to Hao et al. (Hao et al. 2012), who indicated that spherical mesoporous silica nanoparticles are internalized rather via the clathrin-mediated pathway. Interestingly, in our study, the wortmannin inhibits nanoparticles internalization partially, indicating that maybe larger nanoparticles or nanoparticles aggregates are internalized by micropinocytosis (Meng et al. 2011; Vollrath et al. 2013).

**Fig. 7:**
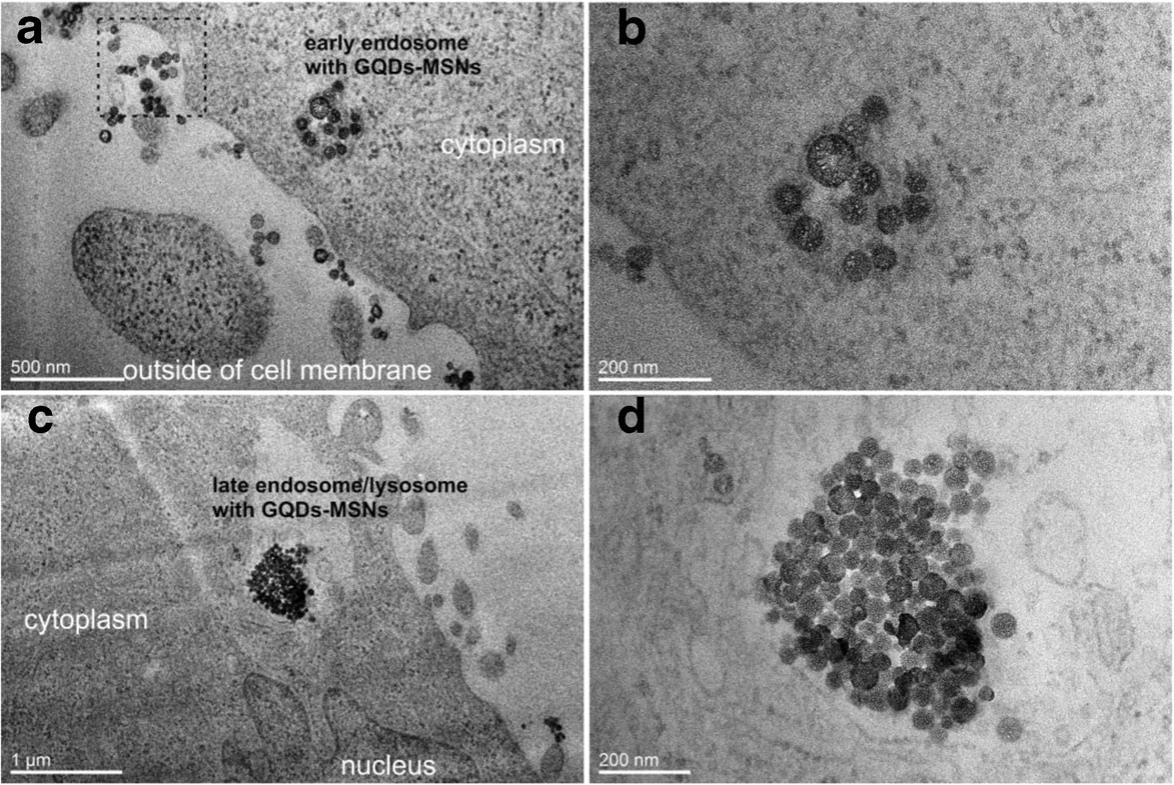
TEM images of HeLa cells treated with GQDs-MSNs showing the cellular uptake and intracellular translocation: **a** endocytosis of nanoparticles through plasma membrane invagination (marked with a square), then GQDs-MSNs are sequestered and dispersed in early endosomes, **b** magnified image of early endosome with nanoparticles, **c** GQDs-MSNs condensed in late endosome/lysosome, **d** magnified image of nanoparticles in late endosome/lysosome

Additionally, to confirm that the uptake of GQDs-MSNs nanocomposites nanoparticles is mediated through the endosome-lysosomal mechanism, TEM analysis was performed (Fig. 7). Obviously, the GQDs-MSNs are uptaken by HeLa cells via their encapsulation into vesicular compartments. The beginning of the uptake occurs by the initiation of plasma membrane invagination, then the nanocomposite nanoparticles are transported to the early endosomes (Fig. 7a), and finally, they are found to be clumped in lysosomes (Fig.7c). Moreover, as presented in magnified images, GQDs-MSNs maintain the spherical and porous morphology. The abovementioned results indicated that the GQDs-MSNs possess both strong enough fluorescence emission and internalization ability, what makes them a promising material for bioimaging and biolabeling.

### Intracellular distribution of doxorubicin

**Fig. 8:**
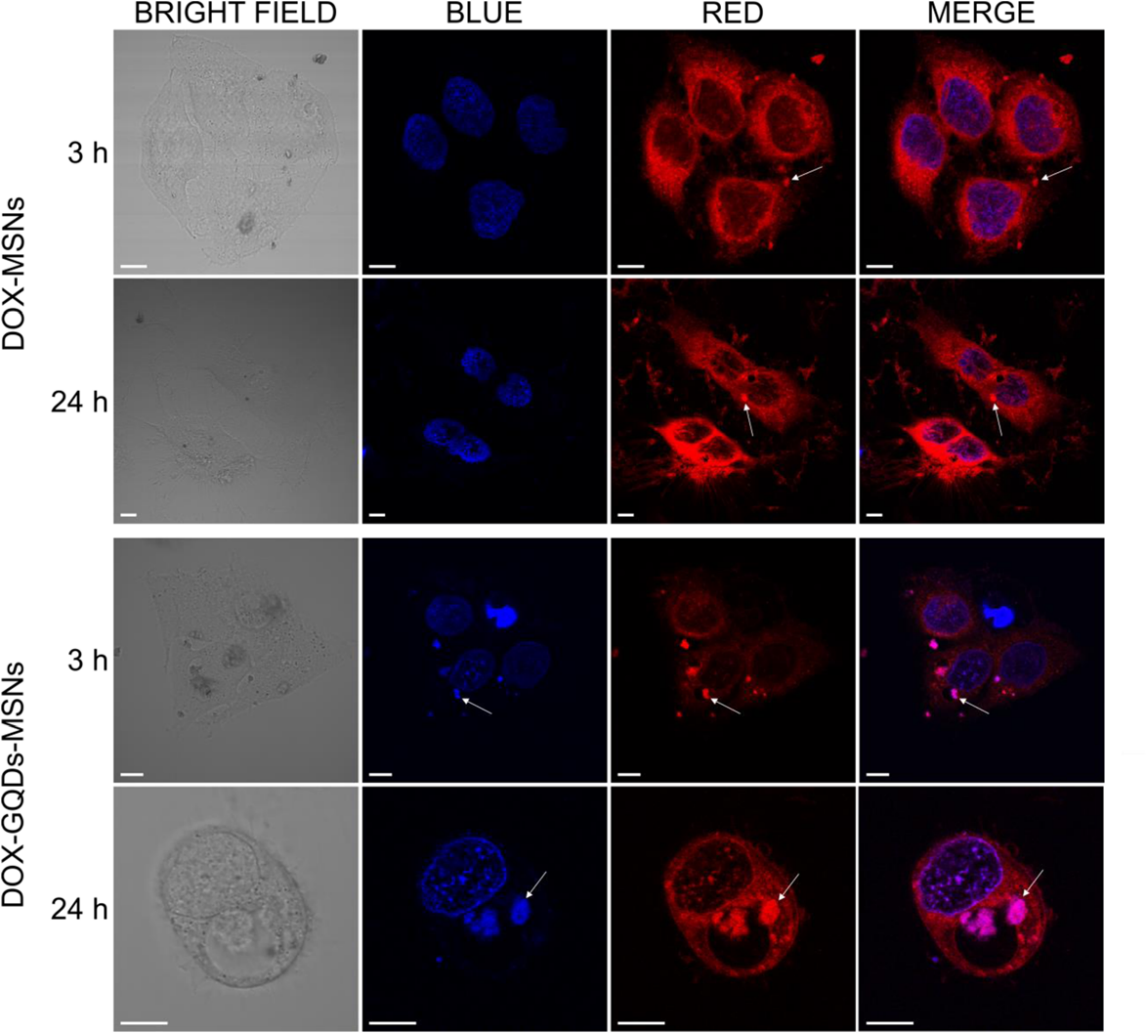
Intracellular distribution of doxorubicin (DOX) delivered and released from DOX-MSNs (upper panel) and DOX-GQDs-MSNs (lower panel) in HeLa cells after 3 and 24 h of incubation with HeLa cells. Channel blue (excitation 405 nm) represents the fluorescence of DAPI-stained nuclei and the fluorescence of GQDs in DOX-GQDs-MSNs, while channel red represents the fluorescence of DOX (excitation 559 nm). Scale bars, 10 μm

Finally, the cell uptake and DOX-MSNs and DOX-GQDs-MSNs and intracellular DOX release after 3 and 24 h of incubation with HeLa cells was also monitored with CLSM. As presented in the Fig. 8, after 3 h of incubation red fluorescence signals from DOX molecules in the DOX-MSNs and GQDs-MSNs nanoparticles can be observed mainly in the cytoplasm, suggesting the endocytosis of the nanoparticles by HeLa cells and DOX release. The observed bright red dots (marked with arrows in Fig. 8) are related to the aggregated carriers, whereas the homogenously spread red fluorescence signal throughout the cytoplasm is related to DOX molecules, which have been released from the nanoparticles within the acidic endosome and have diffused into the cytoplasm. Regarding the DOX-GQDs-MSNs, the use of fluorescent GQDs enables to monitor simultaneously the uptake of the DOX-GQDs-MSNs through channels corresponding to GQDs (mainly blue) and to DOX (red). This is consistent with the imaging results for HeLa cells treated with un-loaded GQD@MSNs presented in Fig. 6. Further incubation up to 24 h with higher dose of DOX-GQDs-MSNs (200 μg/ml) resulted in a marked increase in the releasing of DOX as observed with stronger fluorescent signal (Fig. S6), and these results are in accordance with the DOX-loading and release efficiency. Moreover, cytotoxicity studies indicated that the cytotoxic effect of DOX-GQDs-MSNs is observed for doses above 100 μg/ml in case of HeLa cells**.** Therefore, in case of DOX-GQDs-MSNs, use of higher doses is necessary to induce effects similar to those caused by DOX-MSNs.

In order to investigate the colocalization of the GQDs-MSNs nanocomposite nanoparticles with DOX, the analysis was performed using the Coloc2 tool and the Pearson’s correlation coefficients were found. These coefficients are 0.77 and 0.44 for cells incubated with the 20 μg/ml of DOX-GQDs-MSNs for 3 and 24 h, respectively. In case of the cell exposure to 200 μg/ml of DOX-GQDs-MSNs for 3 and 24 h, found coefficients were 0.39 and 0.37, respectively. These results indicate that colocalization occurs and is more significant in case of shorter time of incubation, whereas longer time of incubation results in the decreased colocalization coefficient. This confirms the time-dependent release of DOX from the GQDs-MSNs nanocomposite nanoparticles.

Summarizing, DOX-GQDs-MSNs nanocomposite nanoparticles are found to be efficient in DOX delivery via their internalization in HeLa cancer cells but also allow for simultaneous real-time optical tracking of the DOX during its delivery and release.

## Conclusions

In this study, we reported on the successful preparation of GQDs-MSNs nanocomposite nanoparticles as an efficient intracellular drug delivery system, but also simultaneously, as fluorescent agent for the optical imaging. Prepared GQDs-MSNs with an average particle size is below 50 nm and high-positive zeta potential form stable suspensions exhibiting excitation-dependent PL behavior. Moreover, they can be easily loaded with doxorubicin chosen as a model drug and show the pH- and temperature-dependent doxorubicin release behavior, due to weakening of the interaction between DOX molecules and GQDs-MSNs. The cytotoxicity assays confirmed that the conjugation of biocompatible GQDs with nontoxic MSNs, leads to the creation of GQDs-MSNs nanocomposite nanoparticles with negligible cytotoxicity, which may serve as a potential drug nanocarriers. Moreover, these assays also confirmed the therapeutic action of delivered and released doxorubicin. Further, the in vitro optical imaging efficacy of cells with GQDs-MSNs, resultant of their cellular internalization via caveloae/lipid raft-mediated endocytosis in HeLa cells, was proven with CLSM and confirmed with TEM imaging. The GQDs entrapment onto mesoporous silica nanoparticles structure resulted in the increasing fluorescent signal in comparison to bare GQDs, which could be related with the immobilization and aggregation effect, but also with their surface charge-related more effective intracellular penetration. Moreover, proposed GQDs-MSNs drug delivery systems enabled the simultaneous real-time optical tracking of the drug during its delivery and release, but also the monitoring of the penetration of the nanoparticles itself. These results indicate therefore that the GQDs-MSNs possess both strong enough fluorescence emission and internalization ability, what makes them a promising material for bioimaging and biolabeling.

## Electronic supplementary material

ESM 1 (DOCX 5862 kb)
