# Supplementary material for: GQDs-MSNs nanocomposite nanoparticles for simultaneous intracellular drug delivery and fluorescent imaging
Source: J Nanopart Res. 2018 Nov 17;20(11):306. doi: 10.1007/s11051-018-4416-y (PMC6244793; doi:10.1007/s11051-018-4416-y)
Supplement: Supplementary file 1 — (DOCX 5862 kb) [file 11051_2018_4416_MOESM1_ESM.docx]

Electronic Supplementary Material

**GQDs-MSNs nanocomposite nanoparticles for simultaneous intracellular drug delivery and fluorescent imaging**

Dorota Flak^a^^[[1]](#footnote-1)^, Łucja Przysiecka^a^, Grzegorz Nowaczyk^a^, Błażej Scheibe^a^, Mikołaj Kościński^a,b^, Teofil Jesionowski^c^, Stefan Jurga^a^

*^a^ NanoBioMedical Centre, Adam Mickiewicz University in Poznań, Umultowska 85, 61-614 Poznań, Poland*

*^b^ Department of Physics and Biophysics, Poznań University of Life Sciences, Wojska Polskiego 38/42, 60-637 Poznań, Poland*

*^c^ Institute of Technology and Chemical Engineering, Faculty of Chemical Technology,*

*Poznan University of Technology, Berdychowo 4, Poznan 60-965, Poland*

~~
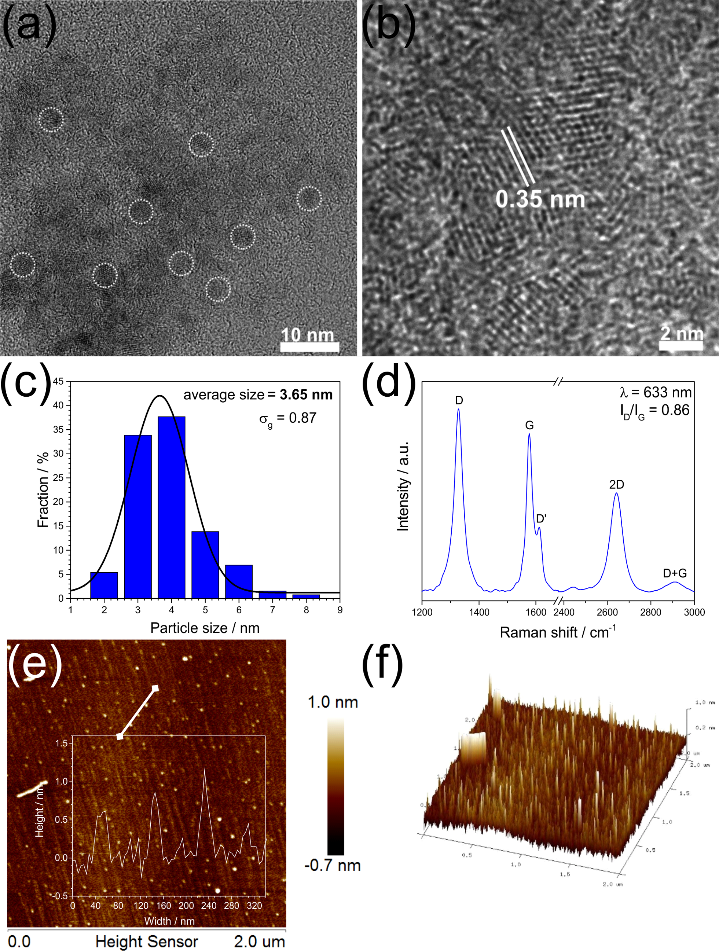
~~

**Figure S1.** The HRTEM images of GQDs: group of GQDs with marked nanostructures of 4 nm diameter (a), magnified images of GQDs with indicated d-spacing of (002) facets characteristic for graphite (b). Particle size distribution (PSD) (c), Raman spectrum (d) and AFM images and inserted height profile (e,f) of hydrothermally prepared GQDs.


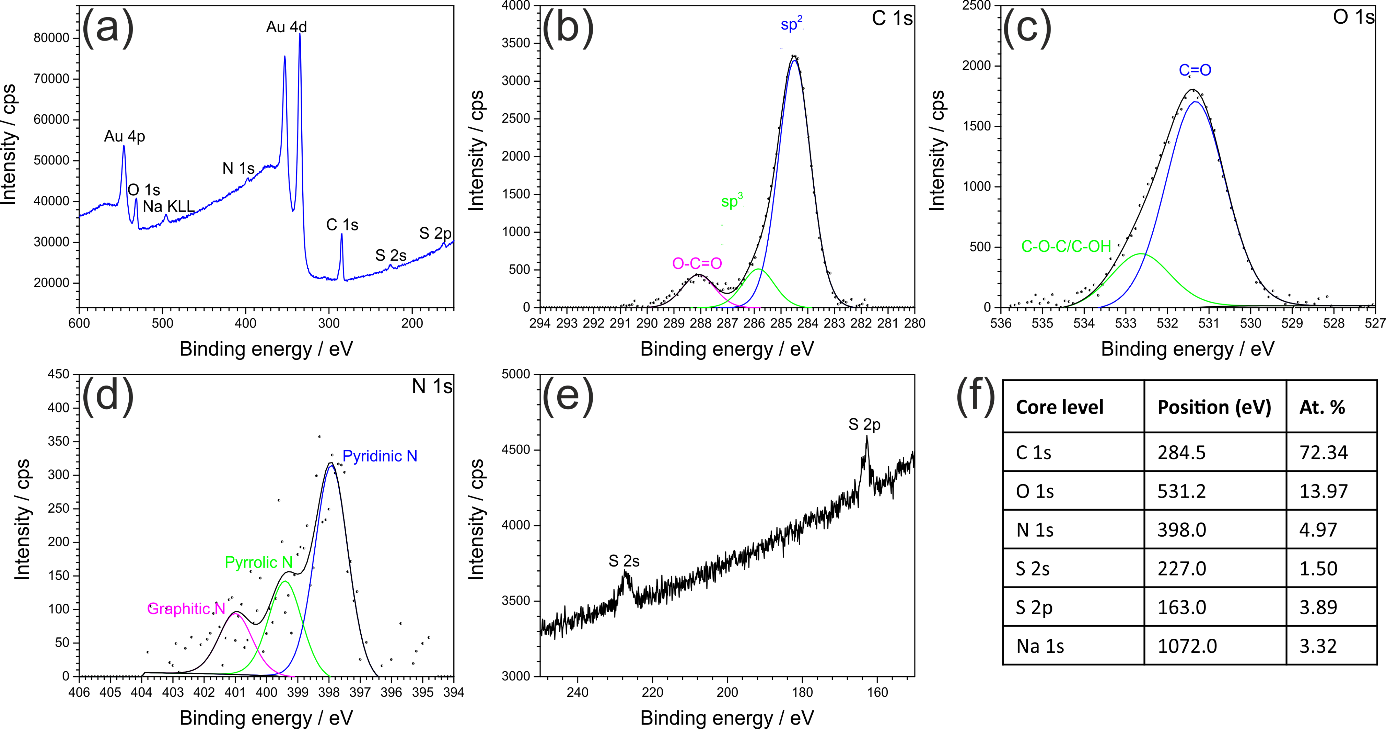


**Figure S2.** XPS spectra of prepared GQDs: survey (a), C1 s (b), O 1s (c), N 1s (d) core level spectra, S 2s and S 2p region (e) and elemental analysis results.


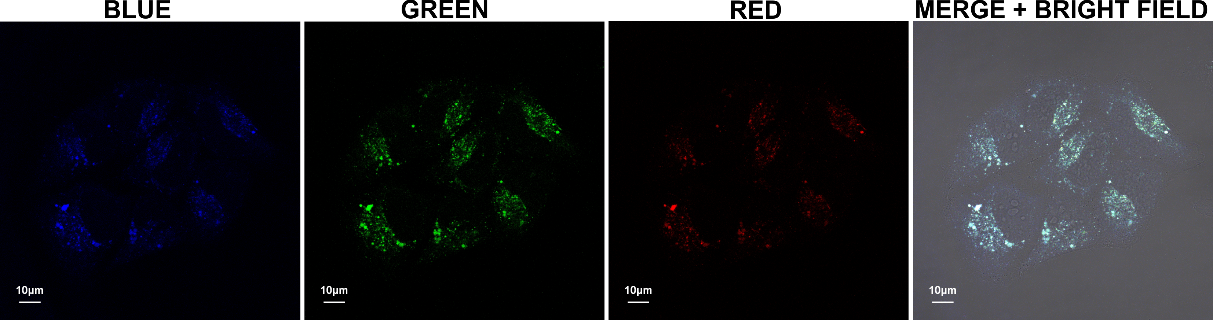


**Figure S3.** Confocal laser scanning microscope images of HeLa cells treated with bare GQDs (50 ug/ml) after 4 h of incubation. Channels blue, green and red represent the fluorescence of GQDs at the excitation 405 nm, 488 nm and 559 nm, respectively (laser power of 3 %).


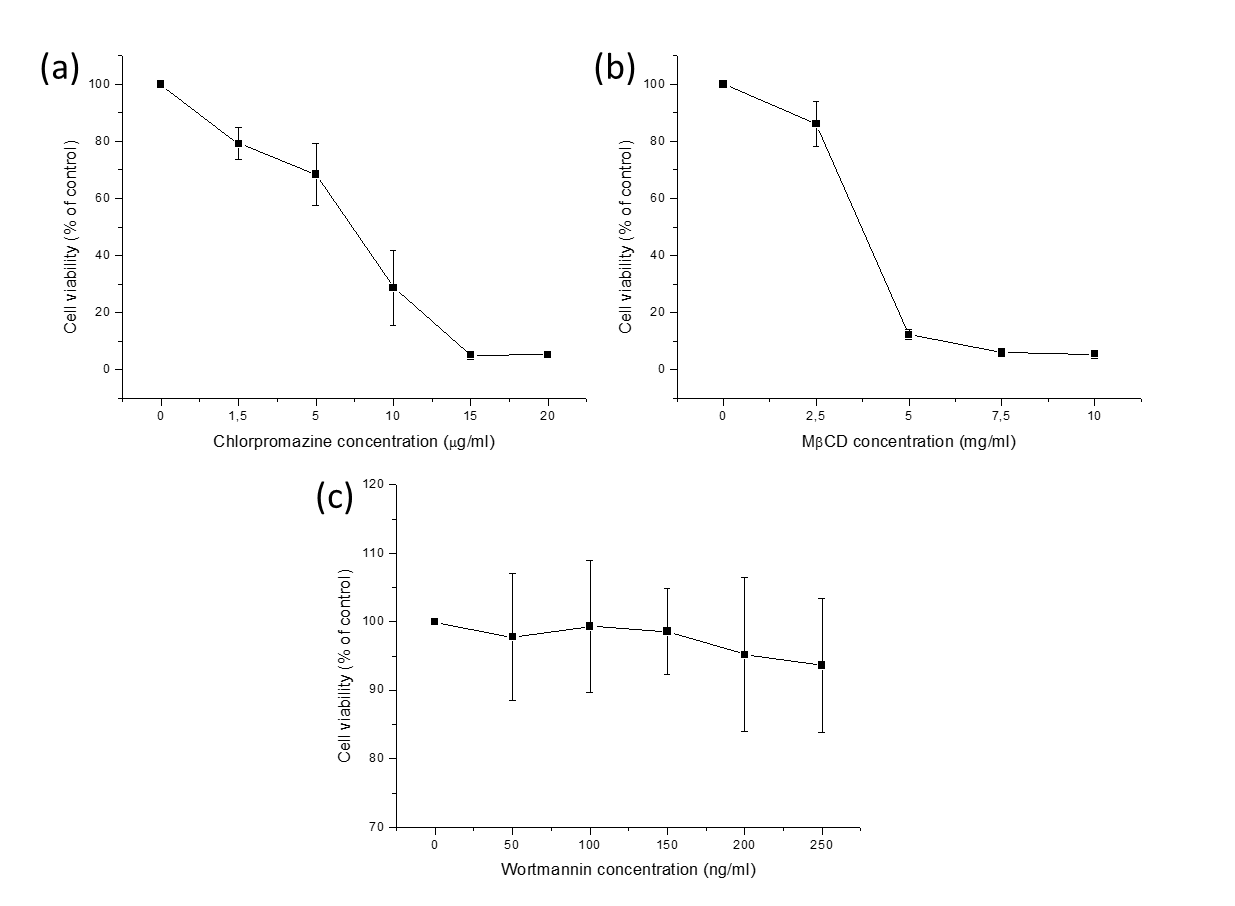


**Figure S4.** *In vitro* cytotoxicity evaluation of chemical inhibitors of cellular uptake: a ) Chlorpromazine, b) Methyl-β-cyclodextrin, c) Wortmannin


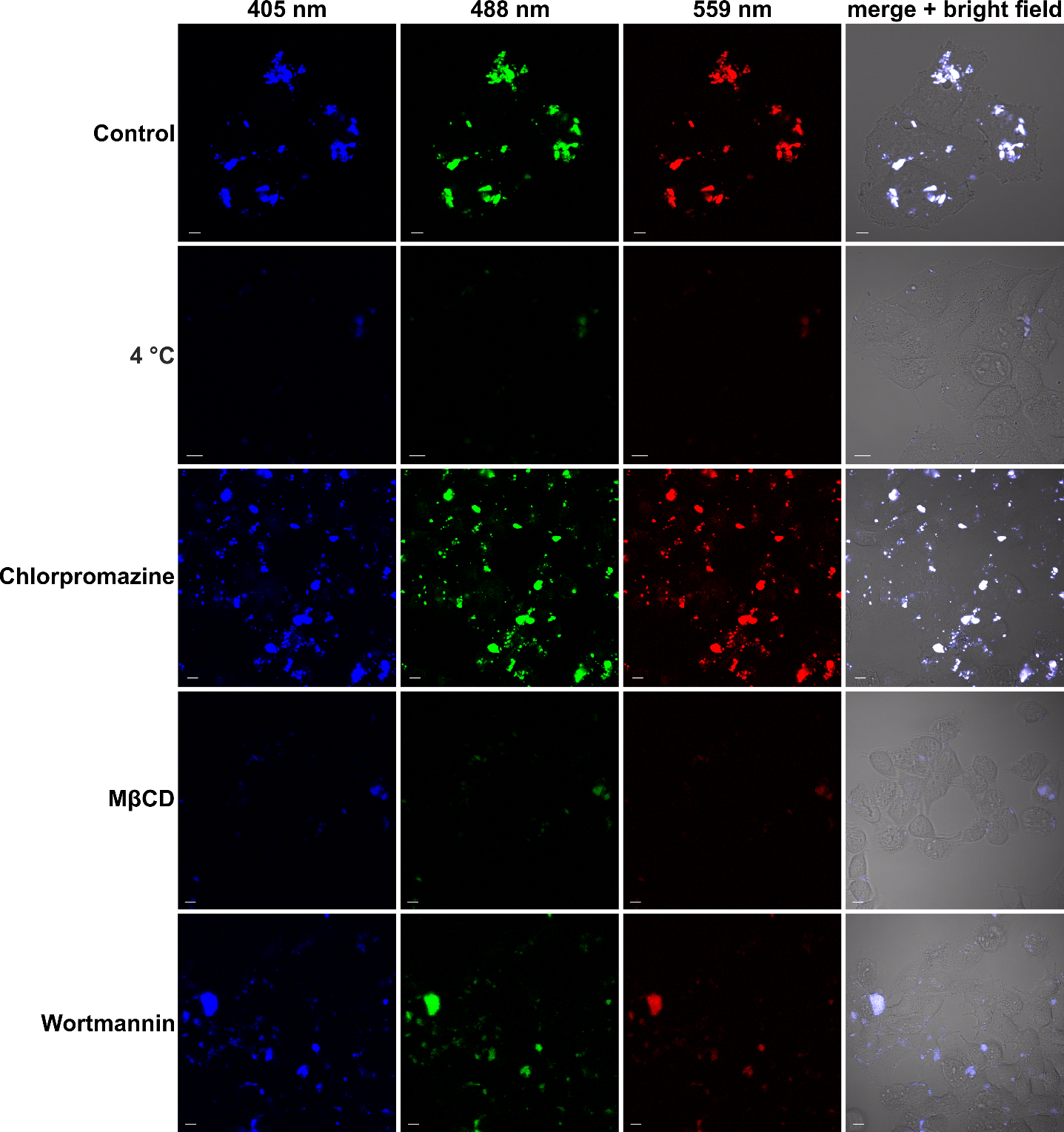


**Figure S5.** CLSM images of intracellular localization of GQDs-MSNs in HeLa cells after treatment with different uptake mechanism inhibitors.


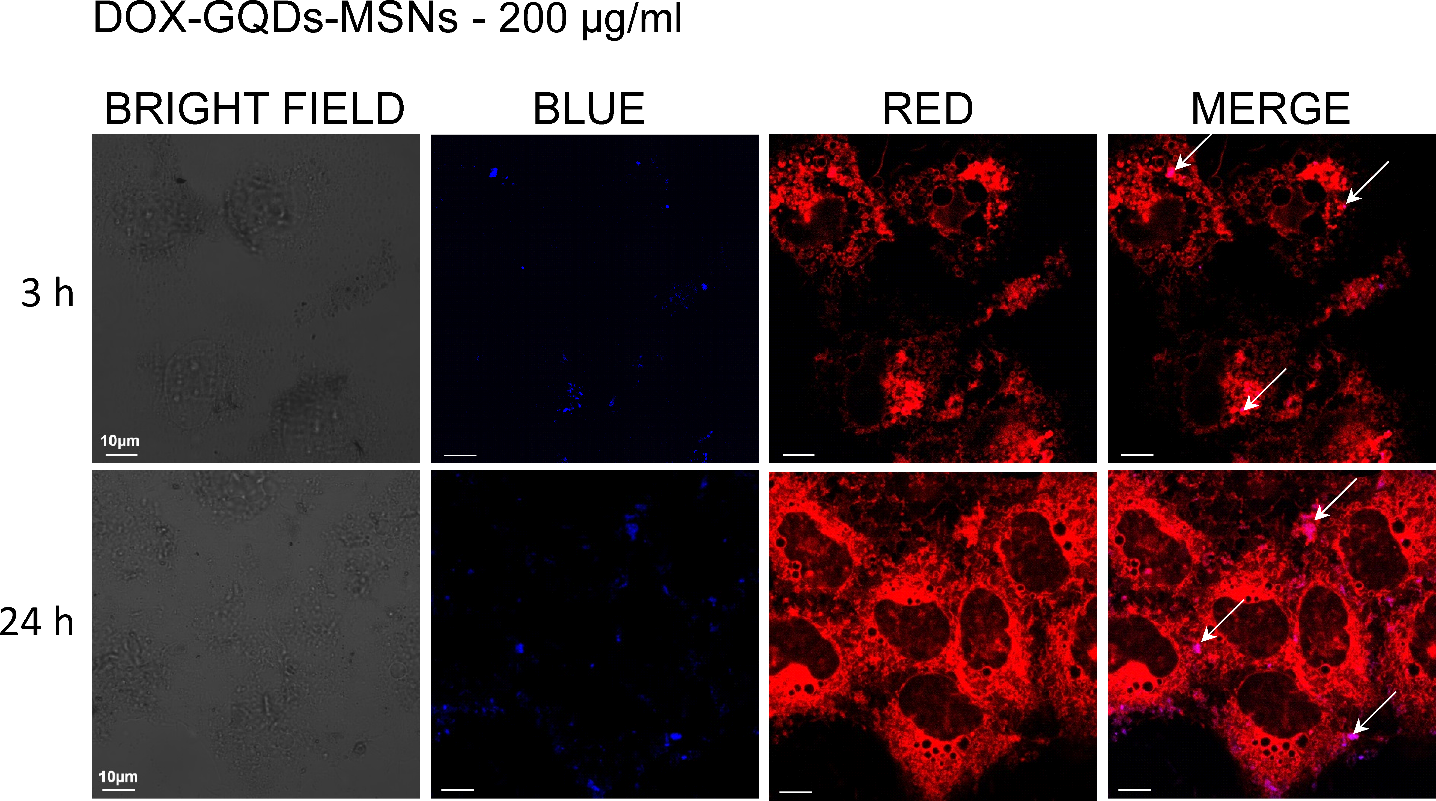


**Figure S6.** Intracellular distribution of doxorubicin (DOX) delivered and released from DOX-GQDs-MSNs (dose 200μg/ml) in HeLa cells after 3 h and 24 h of incubation with HeLa cells. Scale bars - 10 μm.

1. Corresponding Author, email address: [dorfla@amu.edu.pl](mailto:dorfla@amu.edu.pl) (Dorota Flak) [↑](#footnote-ref-1)
